# Surgical Treatment in Post-Stroke Spastic Hands: A Systematic Review

**DOI:** 10.3390/jcm13040945

**Published:** 2024-02-07

**Authors:** Patricia Hurtado-Olmo, Ángela González-Santos, Javier Pérez de Rojas, Nicolás Francisco Fernández-Martínez, Laura del Olmo, Pedro Hernández-Cortés

**Affiliations:** 1 Upper Limb Surgery Unit, Orthopedic Surgery Department, San Cecilio University Hospital of Granada, 18016 Granada, Spain; 2 BIO 277 Group, Department of Physical Therapy, Faculty of Health Science, University of Granada, 18012 Granada, Spain; 3 A02-Cuídate, Instituto de Investigación Biosanitaria, 18012 Granada, Spain; 4 Department of Preventive Medicine and Public Health, San Cecilio University Hospital of Granada, 18016 Granada, Spain; jperezderojas@correo.ugr.es; 5 Escuela Andaluza de Salud Pública (EASP), 18011 Granada, Spain; 6 Instituto de Investigación Biosanitaria ibs, 18012 Granada, Spain; 7 CIBER of Epidemiology and Public Health (CIBERESP), 28029 Madrid, Spain; 8 Rehabilitation Department, San Cecilio University Hospital of Granada, 18016 Granada, Spain; 9 Surgery Department, School of Medicine, Granada University, 18012 Granada, Spain

**Keywords:** stroke, muscle spasticity, upper extremity, hand, operative, surgical procedures, systematic review

## Abstract

**Background**: For more than two decades, the surgical treatment of post-stroke spastic hands has been displaced by botulinum toxin therapy and is currently underutilized. **Objectives**: This article aimed to assess the potential of surgery for treating a post-stroke spastic upper extremity through a systematic review of the literature on surgical approaches that are adopted in different profiles of patients and on their outcomes and complications. **Methods**: Medline PubMed, Web of Science, SCOPUS, and Cochrane Library databases were searched for observational and experimental studies published in English up to November 2022. The quality of evidence was assessed using the Grading of Recommendations Assessment, Development and Evaluations (GRADE) system. **Results**: The search retrieved 501 abstracts, and 22 articles were finally selected. The GRADE-assessed quality of evidence was low or very low. The results of the reviewed studies suggest that surgery is a useful, safe, and enduring treatment for post-stroke spastic upper extremities, although most studied patients were candidates for hygienic improvements alone. Patients usually require an individualized combination of techniques. Over the past ten years, interest has grown in procedures that act on the peripheral nerve. **Conclusions**: Despite the lack of comparative studies on the effectiveness, safety, and cost of the treatments, botulinum toxin has displaced surgery for these patients. Studies to date have found surgery to be an effective and safe approach, but their weak design yields only poor-quality evidence, and clinical trials are warranted to compare these treatment options.

## 1. Introduction

Stroke is the principal cause of permanent disability among adults [1]. More than one-third of stroke patients develop spasticity that requires lifelong medical treatment and increases their dependence on others for daily living activities [2,3]. Upper extremity spasticity frequently results from upper motor neuron damage that is caused by stroke, traumatic brain injury, multiple sclerosis, spinal cord injury, or cerebral palsy [3]. The spastic upper extremity loses the functional position of the hand in space. Patients often have a deformity with internal rotation and shoulder adduction, elbow flexion, forearm pronation, wrist flexion, thumb adduction in palm, and/or flexion of triphalangeal fingers. The deformity can have functional, cosmetic, and/or hygienic repercussions, depending on its intensity [4].

Currently, the first-choice treatment for localized spasticity is the intramuscular injection of botulinum toxin A [5,6,7], although this approach has some drawbacks. Thus, the injection can produce discomfort, the result persists for a maximum of only 3–4 months [8], and it is not useful for muscle or soft tissue contractures [9]. Around 30 years ago, patients with upper extremity spasticity after a first motor neuron injury were treated by surgery, but this option is now rarely considered in spasticity management protocols [7,10] and has been replaced by botulinum toxin therapy. However, no studies have compared the outcomes of these treatment modalities. 

Various surgical procedures can be used to optimize function, reduce pain, and improve hygiene and esthetics in spastic upper extremities [11]. For instance, deformities can be corrected with single-event multilevel surgery, combining releases and elongations of soft tissues, tendon transfers, and joint stabilization procedures [12], or by centering on the nerve as the vehicle of spasticity. In this regard, different authors have proposed hyponeurotization, hyperselective neurectomy [13], and even rhizotomy of the C7 root of the affected extremity, followed by contralateral C7 nerve root transfer [14,15,16], to release the spasticity of the flexor musculature and strengthen weak extensor muscles.

Despite evidence of the long-term effectiveness of surgery in improving the function and hygiene of spastic upper extremities [12,17,18], it is now little used for this purpose [10], hampering evaluation of its true therapeutic potential and the risk of complications. We undertook a systematic review of the literature on surgical approaches that have been adopted in different profiles of patients and on their outcomes and complications, evaluating the quality of the published evidence. The aim was to guide clinical practice and to summarize available evidence for post-stroke patients with a spastic upper extremity who are interested in treatments other than botulinum toxin. 

## 2. Material and Methods

This systemic review and its reporting followed the 2020 Preferred Reporting Items for Systematic reviews and Meta-Analyses (PRISMA) guidelines [19] (Table 1). The review protocol was prospectively registered in PROSPERO with ID CRD42022366686 (www.crd.york.ac.uk/PROSPERO, accessed on 15 November 2022).

## 3. Data Sources and Searches

The Medline PubMed, Web of Science, SCOPUS, and Cochrane Library databases were searched for studies up to November 2022 in accordance with the above protocol, using the following search strategy equation: (hand OR wrist OR thumb) AND (paralysis OR spastic* OR deformity* OR palsy) AND (transfer* OR surgery OR surgical OR neurectomy) AND (stroke OR cerebrovascular OR CVA). The reference lists of selected studies were also examined for relevant articles in a reverse search. Rayyan Systematic Review Screening Software (https://www.rayyan.ai/, accessed on 16 May 2022) was employed to identify and eliminate duplicates.

## 4. Study Selection

The titles and abstracts of retrieved articles were independently screened by two reviewers (AGS, PHO) to select publications meeting the review’s eligibility criteria. A third researcher (PHC) was consulted to resolve cases of disagreement. Inclusion criteria were as follows: observational or experimental design, from case series to clinical trials; evaluation of surgical treatment of post-stroke spastic upper extremity in patients of any age; and publication in English, regardless of the country of origin. Exclusion criteria were review articles, expert opinions, single case reports, exclusive focus on shoulder, cadaver research, qualitative research, and non-availability of whole text.

## 5. Data Extraction and Quality Assessment

The full texts of articles that were selected in the initial screening were reviewed independently by AGS and PHO to decide on their suitability for inclusion and to carry out data extraction and quality assessment procedures. PHC was consulted in cases of disagreement. Articles traced in the reverse search underwent the same process. Data were extracted on the author(s), year of publication, geographic origin, study type, sample size, baseline patient profile and diagnosis, surgical procedure, sample distribution, method of evaluation, efficacy and safety outcomes, and follow-up period. 

The Grading of Recommendations Assessment, Development, and Evaluations (GRADE) was used to evaluate the quality of the evidence as high, moderate, low, or very low [20], and the Cochrane Collaboration tool served to assess the risk of bias. It was not possible to perform a meta-analysis due to the heterogeneity of patient samples, procedures, and outcomes.

## 6. Results 

**Figure 1:**
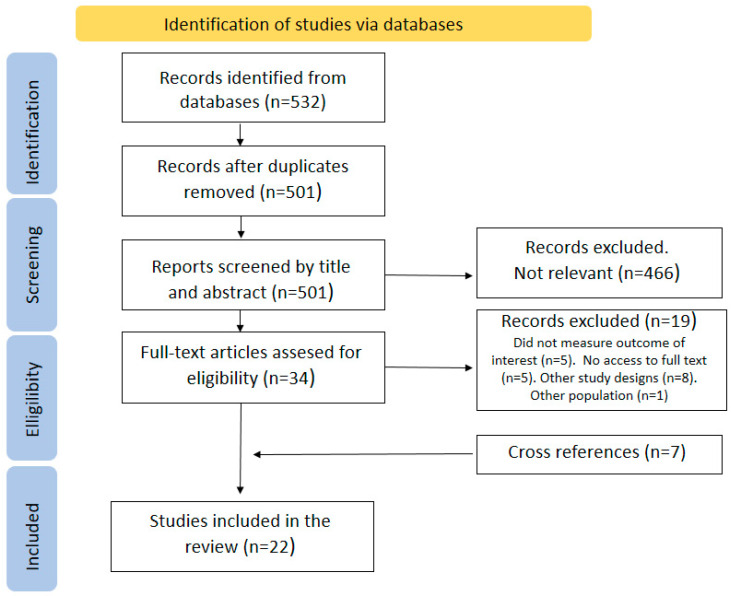
Preferred Reporting Items for Systematic Reviews and Meta-Analyses (PRISMA) flow chart of search results.

The search initially retrieved 501 abstracts (after removal of duplicates), 34 of which met the eligibility criteria. After reading the full texts of the studies, 19 were excluded, but 7 studies were added from the reverse search, leaving a total of 22 studies in the systematic review. Table 2 provides summarized information on the selected studies. Figure 1 depicts a flowchart of the review process. 

### 6.1. Quality of Evidence 

**Figure 2:**
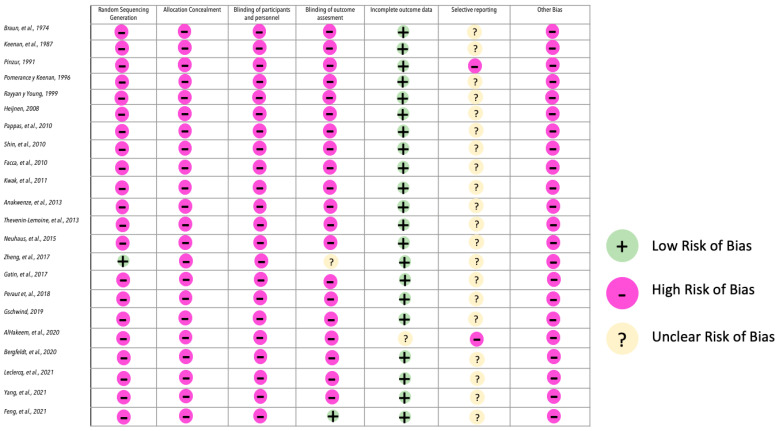
Summary of quality and risk of bias assessment using the Cochrane Collaboration tool [12,16,17,18,21,22,23,24,25,26,27,28,29,30,31,32,33,34,35,36,37,38].

Among the 22 reviewed studies, 19 (86.36%) provided level III evidence, and 21 (95.45%) obtained a low or very low score on the GRADE scale (Table 3) [39,40] (Figure 2). 

Sixteen studies were retrospective case series [12,17,18,21,22,23,24,26,27,28,29,30,31,32,33,37], two were single-center [25] or multi-center [38] retrospective cohort studies, three were prospective case series [34,35,36], and one was a randomized controlled trial [16]. Only three studies included a comparative group, formed by patients who were treated with rehabilitation in two [16,38] and those undergoing a different surgical technique in the third [25]. No studies compared surgery and botulinum toxin treatment. The main sources of bias were a small sample size, a short follow-up period, the absence of a control group, a heterogeneous patient sample, and the partly subjective evaluation of outcomes (Table 3). The mean postoperative follow-up was 21.22 months (range, 6–47.7 months). The follow-up period was one year or shorter in eight (36.36%) of the studies.

### 6.2. Patient Profile

**Figure 3:**
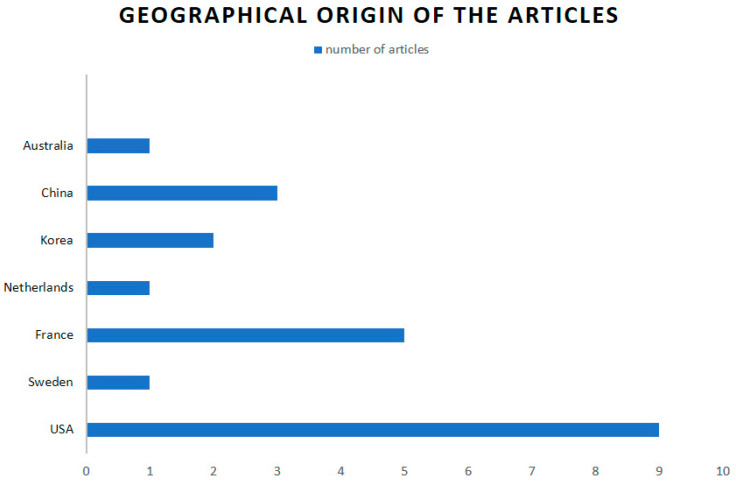
Distribution of the geographic origin of selected articles.

The studies reported on a total of 965 upper extremities in 939 patients. The patient samples were heterogenous in all except two studies [37,38], comprising not only patients with stroke but also those with other etiologies of upper extremity spasticity, including traumatic brain injury and cerebral palsy. Stroke sequelae in the upper extremity was observed in 355 (37.80%) of the patients. The goal of surgery was exclusively hygienic in ten studies [12,16,17,18,24,25,31,32,33], which included a total of 287 hands in 270 patients (i.e., 29.74% of hands in all reviewed studies and 28.75% of patients). Only two articles studied candidates for functional surgery [26,29], reporting on a total of 56 patients (5.96% of patients in reviewed studies). In the remaining studies, the patient sample was mixed, with functional and nonfunctional hands or hands of unspecified status [21,28,30,34,35,36,37,38,41]. None of the reviewed studies stratified their outcomes according to the etiology of the upper extremity spasticity or the patient profile. Overall, 690 males (73.48%) and 249 females (26.51%) were treated. Inadequate data are available to calculate the mean age of the global series, complicated by differences in the etiology of the patients’ spasticity; however, the mean age was <50 years in 16 of the 22 studies. Figure 3 depicts the geographical origin of the reviewed articles.

### 6.3. Types of Surgery 

The most frequently reported surgical approaches (Table 4) were the transfer of *Superficialis*-to-*Profundus* (STP) flexors, muscle–tendon releases, wrist arthrodesis, and peripheral neurectomies. Contralateral C7 nerve root transfer was described in only three studies [16,37,38].

### 6.4. Effectiveness/Efficacy and Safety

Table 5 displays the different evaluations of outcomes, which can be classified as very good, especially for hygiene improvement and pain reduction, with patient satisfaction rates ranging between 65 [28] and 100% [18]. 

Hygiene improvement was reported in 87.5 [21]–100% of operated patients [17,18,22,24,33,42]. Kwak et al. [28] described a reduction in the visual analog scale (VAS) score for pain in a spastic upper extremity from 5.85 pre-surgery to 2.28 post-surgery. Gatin et al. [32] reported on 63 patients with a nonfunctional spastic hand who obtained post-surgical Goal Attainment Scaling (GAS) scores of 1.1 for analgesia, 1.0 for cosmetic appearance, and 1.3 for hygienic conditions, indicating that the outcomes were better or much better than expected. Gschwind et al. [12] studied 38 patients with severe spasticity and nonfunctional upper extremities and found a significant improvement in the Carer Burden Score at three months post-surgery. 

Studies of postoperative functionality changes report statistically significant improvements in the Upper-Extremity Fugl-Meyer scale score (increases of 11–24 points vs. baseline or rehabilitation control group) [16,36,37,38] and in House Scale score [30,31,33] (increases of 0.88–2 points). Two research groups described an improvement in hands from nonfunctionality to a functionality of 18.51–100% [23,30]. Various studies have described a reduction in spasticity of between 0.8 and 2 points on the modified Ashworth scale [16,26,28,29,35,36,37,38], with a persistence of functional improvements and spasticity reduction for 12 to 31 months [28,36]. It was also reported by AlHakeem et al. [34] that gait was improved by spastic hand surgery in three patients. 

No postoperative complications were reported by seven (31.81%) of the reviewed articles [18,24,25,34,36,38,41], and there have been no reports of surgery-related deaths. However, four studies [12,24,26,28] describe postoperative wound infections in between 2.63 [12] and 9.09% [28] of patients. The most frequently reported complications are incomplete correction [27] or deformity recurrence (12.50–27.27% of operated hands) [21,30,31], spasticity relapse (6.66–14.28%) [32,35], unmasking of intrinsic hand muscle spasticity with the emergence of new deformities such as swan neck fingers (9.09–38.46%) [27,30,31,33,42], and finally, prehension weakness due to excessive tendon elongation, observed in 9–20% of patients undergoing tendon lengthening and muscle release [35,42]. Wrist arthrodesis nonunion has been described in between 0% [24,25,27] and 18.18% [31] of cases. 

The only reported complications of peripheral nerve surgery have been mild, although two [25,36] of the seven studies did not provide any data on adverse effects. Paresthesia and dysesthesia were reported in less than 10% of peripheral neurectomies [26,28], and no severe or disabling sequelae were observed after crossed C7 nerve transfer, when the most common complication was pain in the shoulder, back, or extremity at one-month post-surgery (in 58%) that usually disappeared at six months [38]. In donor extremities, fatigue was reported in 41.66%, hand numbness in 44.44%, elbow weakness in 42.66%, wrist extension weakness in 44.44%, and sensory attenuation in 44.44% [16].

## 7. Discussion

We present the first systematic review of studies on post-stroke spastic upper extremities, based on a selection of 22 articles published up to 2022. Systematic reviews and meta-analyses have been performed on the efficacy of botulinum toxin type A in these patients [43,44,45] but not on the efficacy of surgery.

In 2021, Hashemi et al. published [46] a systematic review on the efficacy of surgery in treating spastic upper extremeties of different etiologies, and some study samples contained no patients with stroke (neither CVA nor Stroke was a search term). The larger number of items that were retrieved is attributable to their inclusion of publications in French or Farsi, review articles and updates, and series of patients whose shoulder alone was treated. Outcomes were analyzed as a function of anatomical site (i.e., shoulder, elbow, wrist, hand, or fingers) and main surgical procedure; however, no study included contralateral C7 root transfer, probably the most innovative surgical approach to date. The authors were unable to draw specific conclusions about the efficacy of surgery because of the differences among studies in patient samples and procedures. The same problem of heterogenous populations and the failure to stratify outcomes by the cause of spasticity also limited earlier systematic reviews on thumb-in-palm deformity by Smeulders et al. in 2005 [47] and on peripheral neurectomy by Yong et al. in 2018 [48]. All cases of upper extremity spastic paresis result from upper motor neuron syndrome, but treatment decisions must take account of the etiology and the age and profile of patients [49]. The surgical goal differs between patients whose hands have functional possibilities and those with more severe upper extremity spasticity and nonfunctional hands. Surgical endpoints for the latter group of patients are solely related to hygiene, esthetics, or comfort, and the same evaluation methods cannot be applied. In this regard, Feng et al. (2021) reduced the variability in assessment methods by focusing on quality-of-life changes and patient-reported outcomes [38]. The follow-up period varied among the studies but was generally too short (≤12 months) to confirm the longer-term efficacy of surgery. 

### 7.1. Efficacy and Safety

#### 7.1.1. Superficialis-to-Profundus Tendon (STP) Transfer 

STP transfer was first proposed in 1974 by Braun et al. [21] to improve hand hygiene in patients with spastic clenched-fist deformities of the hand and no volitional control [50]. Although it impairs their prehension capacity, it is performed in patients with stroke sequelae with no expectation of a functional hand from surgery. It is associated with fewer complications in comparison to flexor-origin release, because it requires lesser dissection, and it is less laborious and faster than the selective elongation of all flexor tendons. 

STP transfer must frequently be combined with wrist flexor elongation and arthrodesis [17,27] or peripheral neurectomies [25,27] to improve deformity correction.

Published outcomes have been very good, with an improvement in hygienic conditions in 100% of patients and satisfaction rates of 87 [39]–100% [17,18]. Pain relief was not always evaluated, but the intervention was described as having a beneficial effect against pain in most cases [18,33], and Peraut et al. [33] observed functional improvement on the House Scale in 7 out of 26 patients.

Published complications include over-correction, incomplete correction, deformity by the unmasking of intrinsic spasticity, and partial baseline deformity recurrence. No systemic complications have been reported. Some authors [18,25] observed no complications, while Peraut et al., 2018 [33], reported postoperative deformities through the “unmasking” of intrinsic spasticity in 38.46% and swan neck finger deformities in 23.07%. Recurrences of the deformity (in 12.5%) were only observed by Braun et al. [21].

#### 7.1.2. Tendon and Muscle Release (Including Flexor-Origin Release)

A selective fractioned elongation of wrist and finger flexors or flexor-pronator-origin slide can be performed to improve function in patients with deformity due to extrinsic flexor spasticity who retain volitive control and sensitivity [50]. These procedures may be combined with tendon transfers to improve wrist extension, mainly from the *flexor carpi ulnaris* (FCU) to the *Extensor Carpi Radialis Longus* (ECRL), but only Gatin et al. [32] described this approach for stroke sequelae. Transfers for extension in infantile cerebral palsy involves a risk of reverse postoperative deformities and alteration of the post-prehension release phase [51], and this possibility should also be considered in post-stroke patients.

Very good outcomes have been reported for these techniques, including significant and consistent improvements in rest position, spasticity, pain, and function [32,35]. Function improvement was achieved in >90% of patients, with volitive control after either selective fractioned tendon elongation [42] or flexor-origin release [23]. 

Although AlHakeem et al. described an improvement in gait function in 2020 [34], it was only observed in three patients, and it would be due to the change in positioning of the upper extremity rather than to the surgical procedure per se.

The rate (<30%) and types of local complications [32] that are observed for flexor-origin release were similar to those reported for STP, including prehension weakness (9 [42]–20% [35]), over-correction, incomplete correction, deformity by “unmasking” of intrinsic spasticity (12 [30]–30% [42]), and partial recurrence of baseline deformity (22% [30]). 

Some of these complications may be reduced with the application of surgery under WALANT (Wide-Awake Local Anesthesia No Tourniquet) versus general anesthesia, as recently proposed by Kumar and Ho [52], because it permits the active collaboration and mobility of patients and a more precise calibration, customizing each fractional tendon elongation in real time. The authors observed no under- or over-corrections when adopting this approach.

#### 7.1.3. Wrist Arthrodesis

All reports on post-stroke wrist arthrodesis have involved patients with nonfunctional hands who were treated for hygiene improvement alone [12,17,24,25,27,31]. In spastic patients with no volitive hand control, wrist arthrodesis is more reproducible and long-lasting in comparison to isolated soft tissue procedures [24]. First-row carpectomy is often necessary to place the wrist in a neutral or slightly extended position [41], and other procedures are frequently associated, including STP, carpal tunnel release, thumb-long flexor elongation, and sometimes ulnar nerve motor branch neurectomy to treat intrinsic spasticity [17,52].

Again, published results are very good and report hygienic improvement [17,24,31,53], wrist flexion correction of between 66° [31] and 85° [24], and reduced carer burden [12] in virtually all patients. A screwed compression or neutralization plate with autograft was used as an internal fixation method by all authors except for Rayyan and Young (1999) [24], who employed a structural iliac graft. 

Only local complications have been reported, although these affected one-third of the patients studied by Pomerance and Keenan (1996) [17]. The procedure-specific complication is non-consolidated pseudoarthrosis; however, although a nonunion rate of 13% was observed by Pomerance and Keenan in 1996 [17], more recent articles have not reported this complication. Once more, the procedure can be complicated by the unmasking of intrinsic spasticity and swan neck finger deformity [31,44].

#### 7.1.4. Selective Peripheral Neurotomy

In 1913, partial or selective neurectomy of specific motor nerve fascicles was proposed by Stoffel [54] to improve function in patients with upper extremity spasticity, and the treatment gained in popularity after the publication of the study by Brunelli and Brunelli [55]. The authors initially resected 50% of nerve branches at their muscle insertion points; however, observations of spasticity recurrence due to the “adoption” phenomenon [55] led them to resect 80% of branches or carry out a second neurectomy some months after the first intervention. This procedure has recently been refined [13,56], and partial neurectomy is now performed at the insertion point of each muscle motor branch. 

Neurectomy is most frequently performed in the ulnar nerve motor branch [12,25,27], median nerve recurrent branch [25,28], musculocutaneous nerve [26,36], and the nerves of *Pronator Teres* (PT), *Flexor carpi radialis* (FCR), and FCU [36]. A pre-surgical anesthetic block of peripheral nerves allows for differentiation between spasticity and contracture. The published neurectomy outcomes have been very good, obtaining a significant decrease in spasticity on the modified Ashworth scale, with mean patient satisfaction rates ranging between 64.09 [28] and 83% [36]. Virtually no complications are reported, except for some cases of surgical wound infection. There has been little research on “intrinsic minus” hand deformities, and only Facca et al. [27] described incomplete corrections, observed in around half of patients. Despite expectations related to the adoption phenomenon [55], authors observed no recurrences [28] or only a slight relapse in spasticity, and improvements remained statistically significant at the final follow-up evaluation [26,28,36].

#### 7.1.5. Contralateral C7 Nerve Transfer

Cervical nerve root transfer from the contralateral side has been used to repair brachial plexus root avulsion since 1986 [57]. Variants of this technique include the interposition of a free nerve graft [57], passing the nerve graft through the retropharyngeal and prespinal space instead of the subcutaneous tunnel on the anterior surface of the neck and chest [58], or direct coaptation of the transfer without nerve graft interposition [59]. Different receptor nerves have been utilized in C7 transfer, and Xu et al. were the first to employ the C7 root of the affected side as receptor in a child with spastic paralysis [14]. In 2018, the first clinical trial of contralateral C7 transfer obtained significantly better results compared with rehabilitation [16] in the reduction in spasticity on the modified Ashworth scale and the improvement in function on the Fugl-Maier scale. Functional magnetic resonance imaging (f-MRI) was used to verify the activation of the ipsilateral brain hemisphere with mobility of the affected arm. In 2021, Feng et al. published the largest contralateral C7 transfer trial to date [38] in more than 400 patients, including 168 who underwent the intervention. There was a predominance of patients with stroke in both the surgery and rehabilitation groups, and contralateral C7 transfer achieved significant improvements in spasticity and function (Fugl-Maier scale). The published complications were all mild, including slight weakness, fatigue, soreness, and discomfort on the unaffected side, and they disappeared at 3–6 months post-surgery [16,37,38]. However, this research was conducted in the specific socio-cultural setting of Asia. It may not be so easy to convince patients in many Western settings to transfer nerve elements from the unaffected extremity, on which they may depend for a certain level of function and independence. For this reason, the proposal to transfer these elements from the affected extremity (as both receptor and donor) is of particular interest, avoiding any risk to the healthy arm.

Nerve transfer is an innovative surgical approach to upper extremity paralysis that is well documented in patients with brachial plexus sequalae and is under evaluation for tetraplegic patients; however, it has not yet been described for spastic upper extremities. Waxweiler et al. [60] and Jaloux et al. [61] combined neurectomy with nerve transfer, performing a partial nerve transfer from spastic muscles (elbow flexor, FCR, and PT) to “recipient” motor branches of weak wrist and finger extensor muscles (ECRL, *Extensor Carpi Radialis Brevis*). The aim was to reduce the spasticity of the former and simultaneously activate the latter.

In general, surgery has proved to be an effective treatment option that offers long-lasting results with a low rate of major complications. This raises questions about the status of botulinum toxin as a first-line treatment for regional post-stroke spasticity of the upper extremity [43], given the absence of comparative data on the effectiveness, safety, and economic cost of the two approaches. Beutel et al. [10] investigated upper extremity reconstructive surgery in patients with stroke or TBI in the USA National Inpatient Sample (NIS) database between 2001 and 2012. They reported that 80% of 730,000 new cases of stroke/year survived the acute episode, 76% of the survivors developed spasticity, and 50% of these spastic patients could benefit from surgery [62]. Nevertheless, only 2132 patients underwent surgery during the 12-year study period, i.e., less than 1% of the suitable candidates for surgery, indicating a marked underutilization of upper extremity reconstructive surgery in this patient population.

### 7.2. Other Therapies

The assessment of the therapeutic potential of surgery does not imply an opposition to treatment with toxin or other conservative therapies. The multiple nonsurgical options that are available for the rehabilitation of upper extremity spasticity should be integrated in a multidisciplinary approach to optimize function and prevent deformity in post-stroke patients. Thus, control of baseline muscle tone can be improved by medication, botulinum toxin injection, and chemodenervation, allowing therapists to maximize muscle strengthening, maintain joint integrity, and increase task-specific training [63]. Orthotics can reduce deformity and improve function [64], and radial extracorporeal shock wave therapy (RESWT) has also been proposed for spasticity reduction [65]. Megna et al. studied post-stroke patients with spastic upper extremities and observed a greater spasticity reduction (modified Asworth scale) in patients who were treated with a combination of physical therapy, botulinum toxin injection, and RESWT than in those receiving physiotherapy and botulinum toxin alone [66]. Promisingly, new technologies are developing novel tools for rehabilitation of the spastic hand, notably robotic therapy [67], virtual reality [68], transcranial magnetic stimulation [69], and brain–computer interface systems [70].

### 7.3. Strengths and Limitations of the Review

The main limitation of this review is the poor quality of the scientific evidence that is offered by the studies, largely due to their design and the heterogeneity of patient samples. Furthermore, inadequate information is provided to enable the stratification of types of patients and procedures for comparison, and studies differ in their outcome evaluation methods, which are sometimes highly subjective. Finally, follow-up periods have been too short to evaluate outcomes and complications over the longer term. It was not feasible to search the gray literature, which might possibly have caused some publication bias, and it was not possible to perform a meta-analysis due to the heterogeneity of patient profiles and evaluated outcomes. Strengths of our systematic review include its compliance with the rigorous PRISMA guidelines and its synthesis of the scant available information on an issue of major clinical relevance.

## 8. Conclusions

The results of this review suggest that surgery is a useful, safe, and durable treatment option for post-stroke spastic upper extremities, although most studied patients were only candidates for hygienic improvement. Patients often require an individualized combination of techniques, and there has been renewed interest over the past ten years in procedures that act on the nerve. However, the reviewed studies provide only weak evidence due to their design and heterogeneous patient populations. There is a need for clinical trials to compare surgery and botulinum toxin in the treatment of these patients. The aim is not to exclude one of these approaches but rather to explore how their potential and indications might be integrated within a multidisciplinary treatment protocol in a complementary manner.

## Figures and Tables

**Table 1:** PICO format.

PICO for the research question: Which surgical approaches to adult patients with post-stroke spasticity of the upper extremity are effective in terms of improving their function, care, and quality of life?	
Patient	Adult patients with post-stroke spasticity of upper extremity.
Intervention	Surgical treatment of spastic upper extremity
Comparator	Untreated patients and/or (when available) patients treated with botulin toxin.
Results	Improvement in function; Improvement in pain; Improvement in care; Improvement in quality of life; Complications.

**Table 2:** Summary of articles included in the review.

1st Author (Year)	Study Design	Sample Size: Patients Hands	Etiology of Spasticity	Groups (Surgical Procedure)	Gender	Age (Years)	Geographical Area	Time Since Diagnosis (Months)	Measurement Tools	Results	Complications	Follow-Up (Months)
Braun et al., 1974 [21]	Case series	23 24	CVA (21) TBI (3)	G1: STPTT	12M 12F	49 (23–63)	USA	36 (3–84)	Deformity correction Pain Hygiene	21 satisfactory results (87.5%) 3 unsatisfactory (12.5%)	3 recurrences of deformity (12.5%)	28 (12–30)
Keenan et al., 1987 [22]	Case series	27 27	CVA (6) TBI (20) Anoxia (1)	G1: FLFF	20M 7F	44 (5–62)	USA	45 (7–240)	- A 6-point functional scale for functional hands - Deformity correction for nonfunctional hands	- Functional hands: Improvement in 91%, deterioration in 9% - Nonfunctional hands: 100% improvement	Weak grip by overlengthening (9%) Unmasked intrinsic spasticity (30%)	33 (13–87)
Pinzur, 1991 [23]	Case series	18 18	CVA (13) CP (5)	G1: FOR and other selective tendon lengthening	NR	NR	USA	NR	Pinzur Functional scale [23]	Progression to assistive or independent function: 100%	NR	35 (24–64)
Pomerance y Keenan, 1996 [17]	Case series	14 15	TBI (9) CVA (5)	G1 STPTT+ wrist arthrodesis	5M 9F	46 (26–81)	USA	NR	Hygiene Deformity correction	- Hygiene problem resolution: 100% - Mild under-correction: 26.67% - Mild over-correction: 33.33%	5 complications (33.3%): 2 arthrodesis nonunion and plate mobilization (13.3%) 1 postoperative edema 2 respiratory complications	12 (8–18)
Rayan y Young, 1999 [24]	Case series	9 11	CP (6) CVA (2) BTI (1)	G1: Wrist arthrodesis + 6 associated tendon release with hygienic goals	5M 4F	22	USA	NR	Subjective: - Satisfaction - Care burden Scale improvement Objective: - Union - Deformity correction - 17 tasks—hand function questionnaire	Subjective - Satisfaction: 8 total and 1 partial - Care burden scale: 9 improved Objective: - Union: 9 bone union - Deformity correction: mean of 85% - Secondary functional improvement: face washing, wheelchair propelling, and picking up objects: 90%	No complications reported	32 (12–62)
Heijnen, 2008 [18]	Case series	6 6	CVA	G1: STPTT	6F	54 (36–73)	The Netherlands	60 (48–98)	- Inspection of skin condition - PROM: goniometry (shoulder, elbow, forearm, wrist, and metacarpophalangeal joints) - Muscle tone: Ashworth scale (shoulder, elbow, forearm, wrist, fingers, and thumb) - Hygiene: VAS - Pain: VAS	- Hygiene scored as very good (VAS:8.9) - Full passive opening of all hands - Resting position with flexion in MCP joints (20–60°) and extension of interphalangeal joints - Muscle tone: elbow, wrist and digit flexors improvement of 1–2 on Ashworth scale Pain disappeared in 2 of 3 painful hands. All patients were satisfied.	No complications reported	19 (7–32)
Pappas et al., 2010 [25]	Retrospective cohorts	23 23	CVA (16) TBI (6) Anoxia (1)	Surgery: STPTT + Ulnar motor branch neurectomy+ wrist arthrodesis G1 (n = 11) Surgery without neurectomy of median nerve recurrent Branch G2 (n = 12) Surgery with neurectomy of median nerve	Group 1: 3M/8F Group 2: 5M/7F	48.35 (16–66) Group 1: 52.2 ± 15.7 Group 2: 44.8 ± 14.6	USA	NR	Postoperative intrinsic spastic TIP deformity development	Group 1: 5 of 11 patients developed intrinsic TIP deformity. Group 2: 2 of 12 patients developed intrinsic TIP deformity	No infection No sensation loss	16.1 (6–32)
Shin et al., 2010 [26]	Case series	14 14	CVA (5) CP (5) TBI (3) MS (1)	G1: Selective peripheral neurotomy (musculocutaneous)	10M 4F	37.29 (19–63)	Korea	-	MAS Satisfaction (VAS)	Patients’ mean preoperative MAS score of 3.28 ± 0.12 was improved to 1.71 ± 0.12, 1.78 ± 0.18, 1.92 ± 0.16, and 1.78 ± 0.18 at 3, 6, and 12 months post-surgery and last follow-up. 65% satisfaction.	1 infection 1 transient paresthesia	30.71 (14–54)
Facca et al., 2010 [27]	Case series	15 19	CVA (12) Lewy body disease (1) CP (1) Encephalitis (1)	G1: STPTT + complementary surgical procedures: arthrodesis, tendon surgery, peripheral neurotomy	11M 4F	55 (25–86)	France	116.8 (24–510)	MHS (6–20)	Mean MHS of 13.87 out of 20 pre-surgery vs. 9.67/20 post-surgery. Several imperfect results	2 incomplete thumb openings 2 unmasked intrinsic spasticity 1 wrist hyperextensions	6.13 (3–13)
Kwak et al., 2011 [28]	Case series	22 22	CVA (7) TBI (7) CP (7) MS (1)	G1: selective peripheral neurotomy (median nerve)	15M 7F	39.68 (19–63)	Korea	101 (19–367)	MAS Pain (VAS) Satisfaction (VAS)	Mean MAS score of 3.27 ± 0.46 pre-surgery vs. 1.82 ± 0.5, 1.73 ± 0.7, and 1.77 ± 0.81 at 3, 6, and 12 months post-surgery. Pain improved from 5.85 to 2.28. Satisfaction was 64.09 (30–90)	No recurrences 2 wound infections 1 paresthesia 1 dysesthesia	39.64 (14–93)
Anakwenze et al., 2013 [29]	Case series	42 42	CVA (30) TBI (11) CP (1)	G1: Fractional elbow flexor lengthening	26M 16F	50.9 (21–78)	USA	79.2	Passive and active motion. MAS	Active extension significantly improved (42° to 20°). Active arc of motion increased from 77 to 113°. Significant improvement in MAS recorded post-surgery (2.7 to 1.9).	2 wound infections	14
Thevenin-Lemoine et al., 2013 [30]	Case series	50 54	TBI (25) CP (10) CVA (11) Anoxia (2) Meningo-encephalitis (2)	G1: Flexor-origin slide	35M 15F	32 ± 14 (15–65)	France	NR	Resting position of the wrist Zancolli and House Classifications	Wrist extension improved from −19 ± 35° pre-surgery to 21 ± 20° post-surgery. Significant improvement of 39°. Significant (*p* < 0.01) improvement in Zancolli and House scores. Ten nonfunctional hands became functional.	12 partial deformity recurrences 7 unmasked intrinsic spasticity	26 ± 21 (3–124)
Neuhaus et al., 2015 [31]	Case series	11 11	CVA (5) TBI (4) CP (2)	G1: Dorsal plate wrist arthrodesis	10M 1F	49 (19–78)	USA	240 (48–516)	Radiographic evaluation Deformity correction House score	Radiographic union 9/11 All patients improved appearance. Mean preoperative 66° of flexion changed to 4° of extension position. Mean House score of 2.8 pre-surgery vs. 4.8 post-surgery	2 edema and blisters 3 aggravated thumbs in palm deformity 1 Swan neck finger deformity	14 (3–42)
Zheng et al., 2017 [16]	Randomized controlled trial	36 36	CP (13) TBI (12) CVA (9) Encephalitis (2)	G1: Contralateral C7 transfer + rehabilitation G2: Rehabilitation	36M	Group 1: 27 ± 9 Group 2: 26 ± 8	China	180 ± 108	UEFM MAS (assessment of five joints, each scored from 0 to 5, with higher scores indicating more spasticity) Neurophysiological and fMRI assessment	Mean increase in Fugl-Meyer score for the paralyzed arm of 17.7 in surgery group vs. 2.6 in control group (*p* < 0.001). The smallest between-group difference in spasticity. Improvement in the thumb, with a 2-unit improvement in 6 patients in the surgery group, a 1-unit improvement in 9, and no change in 3. Transcranial magnetic stimulation and fMRI showed connectivity between ipsilateral hemisphere and paralyzed arm.	Paralyzed side: Shoulder or limb pain G1:13/18; G2: 8/18 Donor side: Fatigue 15/18, hand numbness 16/18, elbow weakness 15/18, wrist extension weakness 16/18, sensory attenuation 16/18 No significant differences in sensorimotor functions assessed by neurologic examination between baseline and 12 months post-surgery in nonparalyzed limb.	12
Gatin et al., 2017 [32]	Case series	63 70	CVA (35) TBI (16) Neurodegenerative (6) Anoxia (4) PC (2)	G1: soft tissue surgery Interosseous tenotomy suture-less z plasty of flexor tendon Opening of first web space	40M 23F	51.3 ± 16.2 (24–87)	France	NR	Goal attainment scaling (GAS) transformed into a T score	Mean GAS score increased by 1.3 for hygiene, 1.1 for pain, and 1.0 for appearance	24 complications 7 postoperative edema 6 wound dehiscence 9 hypertonic deformity 1 cardiac failure 1 hardware intolerance	6.2 (1–30)
Peraut et al., 2018 [33]	Case series	26 26	CVA (22) TBI (3) Tumor (1)	G1: STPTT	17M 9F	57 (36–79)	France	NR	Deformity correction by Keenan classification Hygiene scale Pain (VAS) House score	All hands were type V before surgery. Postoperatively, 10 patients had type I and 12 patients had type II hands. Mean House score of all patients increased from 0 to 0.88, functional improvement was observed in seven patients, and hygienic care improvement in 25/26 hands.	10/26 (38.46%) intrinsic deformity 6/26 (23.07%) Swan neck deformity	47.7 (6.6–142.3)
Gschwind, 2019 [12]	Case series	38 45	CVA (12) CP (10) TBI (7) Neurodegenerative (5) Anoxia (3) Encephalitis (1)	G1: Single-event multilevel surgery: tendon, neurectomy, and wrist stabilization	17M 21F	44 (17–83)	Australia	>24	Carer Burden Score	In all cases, the preoperative Carer Burden Score (mean 2.25, range 1.00–3.50) was significantly improved at 3 months post-surgery.	1 death unrelated to surgery 1 pressure sore in elbow 1 wound infection	6 (3–38)
AlHakeem et al., 2020 [34]	Prospective observational study	3 3	CVA (2) CP (1)	G1: FOR and ulnar nerve and carpal tunnel release	1M 2F	48.33 (20–73)	USA	42 (24–60)	Three-dimensional gait analysis before and 3, 6, and 12 months after surgery (Vicon Motion Capturing System)	Gait analysis demonstrated overall improvements in spatiotemporal parameters (cadence and walking speed) and in lower limb kinematics.	No complications reported	12
Bergfeldt et al., 2020 [35]	Prospective observational study	30 30	CVA (13) Spinal cord injury (9) TBI (5) CP (2) Degenerative CNS disease (1)	G1: Tendon lengthening and muscle release.	23M 7F	57 (28–85)	Sweden	96 (12–288)	MAS Resting position and passive and active range of motion Pain (VAS) COPM	Significant improvements in all outcome measures: decreases in spasticity by 1.4 points and VAS by 1.3 points with increases in COPM (performance by 3.4 and satisfaction by 3.6) and in most measures of joint position and mobility	Increased spasticity and pain in 2 patients and hand weakness in 6 patients at 6 months post-surgery	12
Leclercq et al., 2021 [36]	Prospective observational study	42 (13 children) 42	CVA (19) CP (16) Cord injury (3) TBI (2) Tumor (1) Degenerative CNS disease (1)	G1: Selective peripheral neurotomy	27M 15F	14.4 (6.4–17.9) for children 47.2 (20.8–74.2) for adults	France	216 in CP 93. 6 in the other etiologies	Rest position and active and passive range of motion. Ashworth and Tardieu spasticity scale House scores Goal attainment and VAS satisfaction	Effective reduction in spastic tone with no decrease in muscle strength. Comparison between 6 and 31 months showed persistence of improvements. The goal of surgery was reached in 93% of patients at the last follow-up. Mean satisfaction of 8.3/10	No complications	31
Yang et al., 2021 [37]	Case series	2 2	CVA	G1: Contralateral C7 to C7 cross nerve transfer. For the lower limb, contralateral L5 to S1 cross nerve transfer	1M 1F	50 (36–64)	China	252	MAS UEFM MRC grade Barthel Index Hua Shan Grading	At 10 months post-surgery: reduction in MAS score to 1.5; increases in wrist and hand movements, with MRC 3 of 52 post-surgery vs. 28 pre-surgery and Fugl-Mayer score of 62 post-surgery to 51 pre-surgery	Mild soreness and discomfort on the unaffected side that disappeared at 3 months. No long-term complications	10
Feng et al., 2021 [38]	Retrospective multicenter cohort study, China and South Korea	425 425	G1: CVA (102); TBI (32); CP (27); Encephalitis (7) G2: CVA (208); CP (24); TBI (24); Encephalitis (1)	G1: Surgically treated (n = 168) CC7 cross transfer surgery G2: Rehabilitation alone (n = 257)	Group 1: 142M, 26F Group 2: 214M, 43F	Group 1: 35.8 ± 14.8 Group 2: 39.6 ± 14.5	China	Group 1: 85.2 ± 85.2 Group 2: 76.8 ± 79.2	UEFM. MAS Participant reported quality of life questionnaire	Significantly higher change in UEFM score between baseline and 2-year follow-up in the surgery group, which showed significant improvements at all joints	No severe complications or disabling sequelae. The most frequent complication was pain in shoulder, back, or limb in the first month post-surgery (58%) that generally disappeared within 6 months. A total of 194 instances involving the intact hand were reported within 1 month, but all disappeared within 6 months. A total of 244 instances of changes in muscle strength on the intact side	24

NR: not recorded. M: male, F: female; CP: cerebral palsy. TBI: traumatic brain injury. CVA: cerebrovascular accident. CNS: central nervous system. MS: multiple sclerosis; G1: Group 1. G2: Group 2; STPTT: Superficialis-to-Profundus Tendon Transfer; FLFF: fractional lengthening of finger flexors. FOR: Flexor-Origin Release; TIP deformity: Thumb in the palm deformity; MAS: Modified Ashworth Scale; VAS: visual analog scale. PROM: passive range of motion. MHS: Mini Hand Score (Facca et al., 2010) [27]; fMRI: functional magnetic resonance imaging. COPM: Canadian Occupational Performance Measure. MRC: Medical Research Council Grade for motor function. UEFM: Upper-Extremity Fugl-Meyer Scale.

**Table 3:** Quality of evidence and grade of recommendation for studies in the systematic review.

Study	Level of Evidence *	Source of Bias	Quality of Evidence **	Grade of Recommendation ***
Braun et al., 1973 [21]	3	No control; small sample; heterogeneous etiology; partly subjective evaluation method.	Very low	D
Keenan et al., 1987 [22]	3	No control; small sample; highly heterogeneous in etiology, age, and sex; partly subjective evaluation method.	Very low	D
Pinzur, 1991 [23]	3	Selection criteria unreported; no control; small sample; heterogeneous etiology; partly subjective evaluation method.	Very low	D
Pomerance y Keenan, 1996 [17]	3	Selection criteria unreported; no control; small and heterogeneous sample; partly subjective evaluation method; short follow-up.	Very low	D
Rayan y Young, 1999 [24]	3	Selection criteria unreported; no control, very small and heterogeneous sample; partly subjective evaluation method.	Very low	D
Heijnen, 2008 [18]	3	No control; very small sample; partly subjective evaluation method.	Very low	D
Pappas et al., 2010 [25]	2+	Small sample; wide confidence interval; partly subjective evaluation method.	Low	C
Shin et al., 2010 [26]	3	Small sample; heterogeneous etiology; partly subjective evaluation method.	Very low	D
Facca et al., 2010 [27]	3	Selection criteria unreported; very small sample; heterogeneous etiology; partly subjective evaluation method.	Very low	D
Kwak et al., 2011 [28]	3	Small and heterogeneous sample; partly subjective evaluation method.	Very low	D
Anakwenze et al., 2013 [29]	3	Retrospective design; heterogeneous etiology.	Very low	D
Thevenin-Lemoine et al., 2013 [30]	3	Heterogeneous sample in etiology and sex; partly subjective evaluation method; highly heterogeneous follow-up.	Very low	D
Neuhaus et al., 2015 [31]	3	Very small and heterogeneous sample; partly subjective evaluation method; short and heterogeneous follow-up.	Very low	D
Zheng et al., 2017 [16]	1+	Small and heterogeneous sample; short follow-up; males only.	High	B
Gatin et al., 2017 [32]	3	Heterogeneous sample in etiology and sex; very short and heterogeneous follow-up.	Very low	D
Peraut et al., 2018 [33]	3	Small sample; heterogeneous etiology; highly heterogeneous follow-up.	Very low	D
Gschwind, 2019 [12]	3	Small sample; heterogeneous etiology; partly subjective evaluation method; highly heterogeneous follow-up	Very low	D
AlHakeem et al., 2020 [34]	3	Very short and heterogeneous sample.	Low	D
Bergfeldt et al., 2020 [35]	3	Small sample, heterogeneous etiology; partly subjective evaluation method.	Low	D
Leclercq et al., 2021 [36]	3	Heterogeneous etiology; mixture of children and adults; short follow-up.	Low	D
Yang et al., 2021 [37]	3	Very small sample; short follow-up.	Very low	D
Feng et al., 2021 [38]	2+	Retrospective design; heterogeneous etiology; asymmetric sample size and sex of study groups.	Low.	C

* Level of evidence according to the Scottish Intercollegiate Guidelines Network [SIGN]), ranging from 1++ for high-quality meta-analyses, systematic reviews of clinical trials, or high-quality clinical trials with very small risk of bias to 4 for expert opinions. ** GRADE scale for quality of evidence (Aguayo-Albasini et al., 2014) [39], ranging from High, for high confidence in the agreement between real and estimated effect to Very low, for little confidence in the estimated effect, which is highly likely to differ from the real effect. *** Grade of recommendation according to the Scottish Intercollegiate Guidelines Network (SIGN) [40], ranging from A for at least one meta-analysis or clinical trial classified as 1++ and directly applicable to guideline target populations to D for level 3 or 4 scientific evidence or evidence extrapolated from studies classified as 2+.

**Table 4:** Types of surgery and frequency of their application in the reviewed studies.

Type of Surgery	Number of Articles	Number of upper Extremities *	Percentage of Total **	Citations
Superficialis-to-Profundis tendon transfer	6	113	11.70%	Braun et al., 1974 [21]; Pomerance & Keenan, 1996 [17]; Heijnen, 2008 [18]; Pappas et al., 2010 [25]; Facca et al., 2010 [27]; Peraut et al., 2018 [33]
Tendon and muscle lengthening or release	6	198	20.51%	Keenan et al., 1987 [22]; Pinzur, 1991 [23]; Rayyan & Young, 1999 [24]; Anakwenze et al., 2013 [29]; Gatin et al., 2017 [32]; Bergfeldt et al., 2020 [35]
Flexor origin release	3	75	7.77%	Pinzur, 1991 [23]; Thevenin-Lemoine et al., 2013 [30]; AlHakeem et al., 2020 [34]
Wrist Arthrodesis	6	124	12.84%	Pomerance y Keenan, 1996 [17]; Rayyan y Young, 1999 [24]; Pappas et al., 2010 [25]; Facca et al., 2010 [27]; Neuhaus et al., 2015 [31]; Gschwind, 2019 [12]
Selective peripheral neurectomy	6	165	17.09%	Pappas et al., 2010 [25]; Shin et al., 2010 [26]; Facca et al., 2010 [27]; Kwak et al., 2011 [28]; Gschwind, 2019 [12]; Leclercq et al., 2021 [36]
Contralateral C7 nerve transfer	3	206	21.34%	Zheng et al., 2017 [16]; Yang et al., 2021 [37]; Feng et al., 2021 [38]

Types of surgery performed in the reviewed studies. * Upper extremities treated in each study. ** Total = 710 upper extremities.

**Table 5:** Outcome evaluation methods used in the reviewed studies.

Evaluation Method		Citations
Resting position of the extremity; active and passive mobility		Braun et al., 1974 [21]; Pomerance and Keenan, 1996 [17]; Heijnen, 2008 [18]; Pappas et al., 2010 [25]; Anakwenze et al., 2013 [29]; Neuhaus et al., 2015 [31]; Peraut et al., 2018 [33]; Bergfeldt et al., 2020 [35]; Leclercq et al., 2021 [36].
Visual analog pain scale		Heijnen, 2008 [18]; Kwak et al., 2011 [28]; Peraut et al., 2018 [33]; Bergfeldt et al., 2020 [35].
Changes in hygiene and care capacities		Pomerance & Keenan, 1996 [17]; Rayyan & Young, 1999 [24]; Heijnen, 2008 [18]; Peraut et al., 2018 [33].
Modification of spasticity	Ashworth or Modified Ashworth Scale	Heijnen, 2008 [18]; Shin et al., 2010 [26]; Kwak et al., 2011 [28]; Anakwenze et al., 2013 [29]; Zheng et al., 2017 [16]; Bergfeldt et al., 2020 [35]; Yang et al., 2021 [37]; Feng et al., 2021 [38]; Leclercq et al., 2021 [36]
	Tardieu scale	Leclercq et al., 2021 [36]
Functional Scales	Pinzur 1985 functional scale	Pinzur, 1991 [23]
	17 tasks—hand function questionnaire	Rayyan & Young, 1999 [24]
	Mini Hand Score	Facca et al., 2010 [27]
	Zancolli classification	Thevenin-Lemoine et al., 2013 [30]
	House classification	Thevenin-Lemoine et al., 2013 [30]; Neuhaus et al., 2015 [31]; Peraut et al., 2018 [33]; Leclercq et al., 2021 [36]
	Fugl-Meyer Upper-Extremity Scale	Zheng et al., 2017 [16]; Yang et al., 2021 [37]; Feng et al., 2021 [38]
	Canadian Occupational Performance Measure	Bergfeldt et al., 2020 [35]
	Medical Research Council Grade	Yang et al., 2021 [37]
	Hua Shan Grading System	Yang et al., 2021 [37]
	Barthel Index	Yang et al., 2021 [37]
Goal Attainment Scale		Gatin et al., 2017 [32]; Leclercq et al., 2021 [36]
Modification of Care Burden	Care Burden Score	Gschwind, 2019 [12]
Modification of Gait		AlHakeem et al., 2020 [34]
Functional magnetic resonance and electrophysiology studies		Zheng et al., 2017 [16]
Patient evaluation of procedure using a visual analog satisfaction scale.		Shin et al., 2010 [26]; Kwak et al., 2011 [28]; Leclercq et al., 2021 [36]
Participant Reported Quality of Life Questionnaire		Feng et al., 2021 [38]

## Data Availability

Data are contained within the article.
